# Heritability of haemodynamics in the ascending aorta

**DOI:** 10.1038/s41598-020-71354-7

**Published:** 2020-09-01

**Authors:** Kathryn A. McGurk, Benjamin Owen, William D. Watson, Richard M. Nethononda, Heather J. Cordell, Martin Farrall, Oliver J. Rider, Hugh Watkins, Alistair Revell, Bernard D. Keavney

**Affiliations:** 1 Division of Cardiovascular Sciences, Faculty of Biology, Medicine and Health, University of Manchester, Manchester, UK; 2 National Heart and Lung Institute, Faculty of Medicine, Imperial College London, London, UK; 3 Department of Mechanical, Aerospace and Civil Engineering, Faculty of Science and Engineering, University of Manchester, Manchester, UK; 4 School of Engineering, Multiscale Thermofluids Institute, University of Edinburgh, Edinburgh, UK; 5 Division of Cardiovascular Medicine, Radcliffe Department of Medicine, University of Oxford, Oxford, UK; 6 Division of Cardiology, Chris Hani Baragwanath Hospital, Soweto and the University of Witwatersrand, Johannesburg, South Africa; 7 Population Health Sciences Institute, Faculty of Medical Sciences, Newcastle University, International Centre for Life, Newcastle upon Tyne, UK; 8 Wellcome Centre for Human Genetics, University of Oxford, Oxford, UK; 9 Manchester University NHS Foundation Trust, Manchester Academic Health Science Centre, Manchester, UK

**Keywords:** Biomedical engineering, Genetics

## Abstract

Blood flow in the vasculature can be characterised by dimensionless numbers commonly used to define the level of instabilities in the flow, for example the Reynolds number, *Re*. Haemodynamics play a key role in cardiovascular disease (CVD) progression. Genetic studies have identified mechanosensitive genes with causal roles in CVD. Given that CVD is highly heritable and abnormal blood flow may increase risk, we investigated the heritability of fluid metrics in the ascending aorta calculated using patient-specific data from cardiac magnetic resonance (CMR) imaging. 341 participants from 108 British Caucasian families were phenotyped by CMR and genotyped for 557,124 SNPs. Flow metrics were derived from the CMR images to provide some local information about blood flow in the ascending aorta, based on maximum values at systole at a single location, denoted *max*, and a ‘peak mean’ value averaged over the area of the cross section, denoted *pm*. Heritability was estimated using pedigree-based (QTDT) and SNP-based (GCTA-GREML) methods. Estimates of Reynolds number based on spatially averaged local flow during systole showed substantial heritability (${\hbox {h}}^{2}_{Ped} = 41\% \,[\hbox {P}=0.001]$, ${\hbox {h}}^{2}_{SNP} = 39\%\, [\hbox {P}=0.002]$), while the estimated heritability for Reynolds number calculated using the absolute local maximum velocity was not statistically significant (12–13%; $\hbox {P}>0.05$). Heritability estimates of the geometric quantities alone; e.g. aortic diameter (${\hbox {h}}^{2}_{Ped} = 29\%\, [\hbox {P}=0.009]$, ${\hbox {h}}^{2}_{SNP} = 30\%\, [\hbox {P}=0.010]$), were also substantially heritable, as described previously. These findings indicate the potential for the discovery of genetic factors influencing haemodynamic traits in large-scale genotyped and phenotyped cohorts where local spatial averaging is used, rather than instantaneous values. Future Mendelian randomisation studies of aortic haemodynamic estimates, which are swift to derive in a clinical setting, will allow for the investigation of causality of abnormal blood flow in CVD.

## Introduction

Chronic abnormal blood flow in the aorta, imparting damage to the vessel wall over time, has been associated with cardiovascular diseases (CVD) such as aortic stenosis^1^, aortic dissection, and the growth of aortic aneurysms; both thoracic and abdominal^2,3^. The extent to which the association is causal in CVD remains unclear, and investigation requires systematic harvesting of blood flow data. Phase contrast magnetic resonance (MR) imaging offers a verified means of measuring blood flow rate in the great and peripheral arteries based on the phase-shift of magnetic moments acting on particles as they move through a magnetic field gradient^4,5^, with a generally accepted accuracy of within 5%. While these measurements have some limitations, MR images are increasingly used to generate accurate patient-specific geometries for use in computational fluid dynamics (CFD), to enable a more detailed investigation of the haemodynamics^6–8^, although the process of segmentation, preparation and iterative solution can be time-consuming and resource intensive.

While CFD-derived metrics may at some point be applicable in genetic studies, we sought to investigate an approach potentially applicable to genome-wide association studies (GWAS) which typically require thousands of participants. Non-dimensional flow metrics are readily obtainable from pre-exiting MR image data, derived by combining basic geometric measurements with the flow rate. Calculated as the ratio between inertial and viscous forces in a flow, the Reynolds number, *Re*, is a universally accepted metric to describe the level of turbulence present in a flow. The combination of MR-derived flow data and patient-specific CFD modelling has led to increased interest in assessing haemodynamics and its impact, in particular on vessel walls. Aside from the Reynolds number, other quantities such as the Strouhal and Womersley numbers, which describe the oscillatory and pulsatile nature of the flow respectively, have also received attention^9^. Other efforts to quantify levels of turbulence have been made from the data processing side, by employing the intravoxel velocity standard deviation to approximate local levels of turbulence^10^, however research in this area is still ongoing^11^.

**Figure 1:**
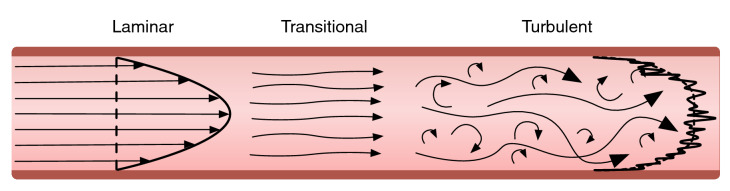
Laminar (left), transitional (middle) and turbulent (right) blood flow: arrows indicate direction of fluid flow. During laminar flow fluid moves in parallel layers without disruption between the layers. During turbulent flow mixing occurs between the layers creating chaotic flow velocities with variations in gradient. The right side of each figure show the velocity profile across the diameter, averaged in time and at a given instance in time.

The flow of blood through vessels is pulsatile in nature, which for adult humans leaves the heart with a velocity at peak systole in the range $$150<U<175 \, \hbox {cm/s}$$, via the ascending aorta of diameter $$3<D<4 \, \hbox {cm}$$^12^. Using standard values of blood viscosity and density, as $$\mu =3\times 10^{-3}\, \hbox {Ns/m}$$^13^ and $$\rho =1050$$$${\hbox {kg/m}}^3$$ respectively, this indicates a Reynolds number, $$Re=\rho .U.D/\mu $$, in the range of 15,000–20,000. This is well beyond the classical threshold between orderly, ‘laminar’ flow and fully turbulent flow, which for carefully controlled conditions was originally reported by Reynolds himself to be around 2,100–4,000^14^. The transition between laminar and turbulent flow with corresponding flow characteristics is illustrated in Fig. 1. Due to the pulsatile nature of the flow, the Reynolds number will vary from zero up to a maximum at peak systole, and so the flow can be expected to cycle through laminar, transitional and turbulent states before returning to laminar in diastole. The flow in the ascending aorta is clearly very different to the controlled transition from laminar to turbulent flow in pipes, but it is nevertheless a direct measure which simultaneously incorporates information about both the geometry and flow rate in the aorta.

The extent to which blood flow in the ascending aorta is fully turbulent in the classical definition of the term is a subject of some debate^15^, and is difficult to measure. Nevertheless, some of the key characteristics of turbulent flow are present; a 3D unsteady, flow characterised by strong vortical motion which induces rapid mixing across the cross-section of the flow. This was found in recent MR-based haemodynamic observations^16,17^. It is therefore logical to expect that these characteristics will be amplified or reduced in a manner proportional to the locally-computed values of the Reynolds number, which is the hypothesis motivating the present study. The questions that this study seeks to answer are the following. Is a locally-computed estimate of Reynolds number derived from historical MR imaging influenced by genetic factors? Does the inclusion of flow information in this way lead to a useful metric when compared to common alternatives?

Wall shear stress arises at a vessel wall due to the transverse motion of an adjacent fluid and is known to be highly sensitive to flow conditions and the level of unsteadiness therein. Mechanosensitive genes have been identified in mouse models^18^ and in human endothelial cells in vitro^2,19^. Shear stress response elements have been found in the promoters of many genes with roles in CVD and aneurysm incidence (such as *PDGF*-$\beta$, *tPA, ICAM-1, VEGR, eNOS,* and *TGF*$\beta$*1*)^20^. For a fixed cycle, a cardiac output with a larger Reynolds number at peak systole can be expected to exhibit a greater unsteadiness, with a higher proportion of vortical flow. In this scenario the interaction between blood and the vessel wall changes, leading to a change in wall shear stress (WSS) profiles which may play a role in the development of CVDs such as atherosclerosis^21,22^. While there is some divided opinion on the matter, the evidence does point more towards a link between high levels of oscillatory shear and an increased risk of CVD^23^, which may also suggest correlation between risk in the ascending aorta with Reynolds number.

We are not aware of any previous heritability estimates of *Re*-based haemodynamics of the vasculature, particularly those which can be linked to peak systole at a given location, i.e. in the ascending aorta. As such we have made use of pre-existing MR data to investigate the heritability of several different haemodynamic metrics in the thoracic ascending aorta in a family-based cohort, to estimate the influence of genetic factors on aortic blood flow characteristics. We show that Reynolds number based on locally-averaged data is significantly heritable in this family-based cohort whereas a Reynolds number based on the maximum reported velocity alone is not. Future large-scale studies may discover the genomic loci influencing the variation in these blood flow traits. Furthermore, such loci can be analysed through Mendelian randomisation to assess for a causal role in cardiovascular abnormalities and disease risk.

## Methods

### Family recruitment

248 families (1,425 participants) were recruited between 1993 and 1996 for a quantitative genetic study of hypertension and other cardiovascular risk factors, and selected via a proband for essential hypertension (secondary hypertension was excluded using standard clinical criteria)^24^. Probands were recruited from the hypertension clinic at John Radcliffe Hospital, Oxford, UK or via their family doctors. Included families were U.K. residents of self-reported white ethnicity and were required to consist of 3 or more siblings quantitatively assessable for blood pressure if one parent of the sibship was available for blood sampling, or 4 or more siblings if no parent was available. First, second and third degree relatives were then recruited to assemble a series of extended British families. DNA from the blood sampling during this phase of recruitment was extracted from whole blood by standard methods. Genotyping was performed using the Illumina 660W-Quad chip that includes 557,124 single nucleotide polymorphisms (SNPs).

In 1997–2000, family members were invited to re-attend for phenotyping using ECG and Echocardiogram; results from that phenotyping exercise have been previously published^25^. In 2007–2010, surviving members of the cohort were invited to participate in cardiac MRI phenotyping, the CMR data that is analysed in this study. 341 participants from 108 families attended. The collection protocol obtained ethical clearance from the Central Oxford Research Ethics Committee (ethics application number: 06/Q1605/113) and it corresponds with the principles of the Declaration of Helsinki. Written informed consent was obtained from all participants.

### CMR imaging and analysis

**Figure 2:**
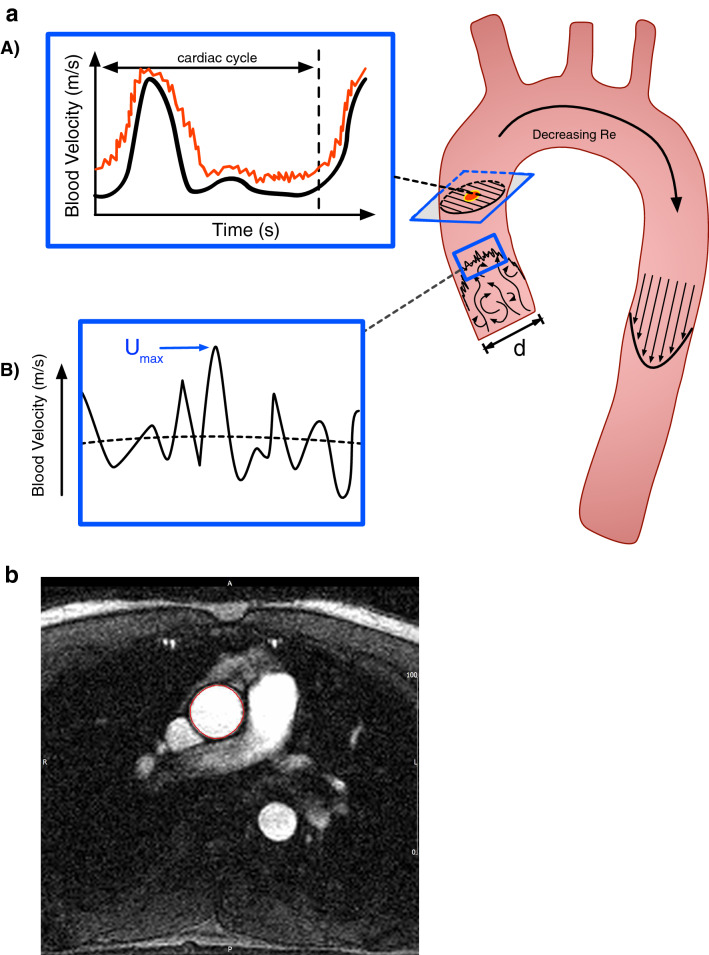
Part a: (**A**) Mean (black) and maximum (red) blood flow velocity across a cardiac cycle. (**B**) $U_{max}$ is the maximum value of instantaneous velocity (solid line) at any location of the imaging plane whereas $U_{pm}$ is the maximum mean velocity (dashed line). Part b: Magnitude image demonstrating contouring of ascending aorta (red outline).

CMR was performed on a 1.5 Tesla scanner (Sonata; Siemens Medical Solutions, Erlangen, Germany) with a dedicated six-channel phased array surface coil. Imaging was performed during an end-expiration breath hold in order to minimize diaphragmatic motion. Scout images in axial, sagittal, and coronal planes were used to localise cardiac position within the thorax and to plot flow sequences. Phase contrast flow-encoded gradient echo sequences were acquired in breath-hold at end-expiration with an axial slice placed perpendicular to the mid ascending aorta at the level of the right pulmonary artery. Scan parameters are as follows: TE/TR $=$ 2.8/11 ms, Venc $=$ 200 cm/s, field of view $320\times 220$ adjusted for each patient, pixel spacing $1.25 \times 1.25$ mm, slice thickness 5 mm, temporal resolution 12 ms. Commercially available software (CVI42 v.5.3.4, Circle Cardiovascular Imaging Inc., Calgary, Canada) was used for analysis, the outline of the ascending aorta was traced manually as shown in Fig. 2.

### Approximate haemodynamics taken from CMR images

A range of canonical flow metrics were analysed. Flow is generally classified by its Reynolds number, *Re*, a dimensionless number representing the ratio of inertial and viscous forces^14^. The value of *Re* for a given flow is dependent on how each of the component quantities is defined. In order to compare flows these definitions must be consistent, for example, in this case the characteristic length of a pipe is the diameter rather than the radius. Furthermore, *Re* can essentially be calculated for different temporal and spatial frames as required and appropriate; for instance it could be based on the peak flow value or the flow averaged over a cross section of the vessel. For CMR images used in this study we test both of these definitions for *Re*, as described in Table 1.

**Table 1:** Definitions and time scales of haemodynamic metrics included in the genetic analyses.

Quantity and formula	Description
General definition $\mathbf {Re}=\frac{\rho U L}{\mu }$	Where $\rho$ is the fluid density, *U* is the fluid velocity, *L* is the characteristic length, and $\mu$ is the dynamic viscosity
Peak Mean Re $\mathbf {Re_{pm}}=\frac{U_{pm} D}{\mu }$	The maximum average Reynolds number across a cross section of the aorta at any point during the cardiac cycle, i.e. generally occurring at systole
Max Re $\mathbf {Re_{max}}=\frac{\rho U_{max} D}{\mu }$	The maximum Reynolds number at any time during the cardiac cycle, at a single position in the ascending aorta

In both cases of *Re*, *D* is the diameter of the aorta for the cross-sectional plane selected in the image, while blood density $\rho$, and blood viscosity $\mu$, take standard values.

The following list provides an overview of the metrics we have computed, and is accompanied by visual representation in Fig. 2. Each of these metrics can be calculated from clinically available data in a straightforward fashion:

1. *Aortic diameter (D)* Measured diameter of the ascending aorta at the cross-sectional plane where flow data is measured.
2. *Max velocity* (${U_{max}}$) the maximum velocity at any point on the cross-sectional plane, at any point in the cardiac cycle i.e. usually but not always at systole. Blood flow velocity measurement is based on the principle that magnetic field gradients introduce a phase shift in the MRI signal arising from the flowing proton spins that is proportional to blood flow velocity. The interested reader is directed to more detailed literature on this subject^26,27^.
3. *Peak mean velocity* (${U_{pm}}$) the mean blood velocity across the cross-sectional plane of the aorta, taken at systole. This is calculated through averaging velocity measurements within the defined cross-sectional contour shown in Fig. 1.
4. *Max flow Reynolds number* (${Re_{max}}$) makes use of the maximum velocity at a single location at any point during the cardiac cycle, $U_{max}$ along with the measured aortic diameter.
5. *Peak mean Reynolds number* (${Re_{pm}}$): instead uses the mean velocity at systole $U_{pm}$, along with the measured aortic diameter.

### Statistical analyses

The traits were adjusted for the effect of covariates by stepwise multiple linear regression in SPSS (version 25). Age, ${\hbox {age}}^{2}$, sex, BMI, clinical systolic blood pressure, and a binary trait describing proband ascertainment status (hypertension) were investigated as covariates of the haemodynamic metrics. Included covariates in the final model had a significance level of ${P} <0.05$, shown in Table 2. Residuals from the covariate-adjusted regression models were standardised to have a mean of 0 and variance of 1. Outliers outside three standard deviations from the mean were excluded.

**Table 2:** Covariates included in the final adjustment model for each trait.

Trait	Identified covariates
Cross sectional area (${\hbox {mm}}^2$)	Sex, age, BMI, hyper
Aortic diameter (m)	Sex, age, BMI, hyper
$U_{pm}$ (m/s)	Sex, age, BMI, hyper
$U_{max}$ (m/s)	Sex, age, BMI
$Re_{pm}$	Sex, age, BMI
$Re_{max}$	Sex, ${\hbox {age}}^{2}$, BMI, BP

BMI, body mass index; hyper, a binary trait representing hypertension status; BP, clinical systolic blood pressure.

### Genetic analyses

Routine genotyping QC was undertaken using PLINK^28^ (version 1.9), to exclude SNPs with low genotyping rates ($<95\%$), individuals with low genotyping rates ($<95\%$), rare SNPs with low minor allele frequency ($<1\%$), and those that were identified as Mendelian inconsistencies. Individuals with outlying average heterozygosity were removed (0.31–0.33 included), as were SNPs that failed checks of Hardy–Weinberg Equilibrium ($<1\times 10^{-8}$). Gender checks and relatedness agreed with reported status and population stratification was assessed via principal components analysis with the 1,000 Genome Project^29^, which confirmed all participants were of European/CEU origin.

Following the initial quality control, 503,221 autosomal SNPs from 1,219 individuals were available to inform imputation. Imputation was performed through the Michigan Imputation Server (version v1.0.4)^30^, specifying pre-phasing with Eagle2 (version 2.3)^31^ and imputation by Minimac3 using the European population of the Human Reference Consortium^32^ (version hrc.r1.1.2016). Following imputation, duplicate SNPs, and SNPs with ${\hbox {R}}^2 < 0.8$ were removed to retain high quality imputed SNPs, and the genotyped SNPs were merged with the imputed SNPs. Quality control was undertaken for the 305 individuals with both genotyping and CMR data available, as per the following standards; –mendel-multigen, –geno 0.05, –maf 0.05, –hwe 1e-8, –mind 0.05, to a final count of 5,272,802 SNPs.

Pedigree-based heritability was estimated using QTDT software^33^ (version 2.6.1) specifying the -we and -veg options to compare an environmental only variance model with a polygenic and environmental variances model. A complementary estimation of heritability was undertaken using GCTA-GREML software^34^ (version 1.26.0) on the genotyping data. A genetic relationship matrix was created from the genotyping data and the –reml command was used to estimate variance of the traits explained by the genotyped SNPs. Linear mixed modelling approaches were used to account for family structure. GWAS were undertaken for the metrics using GCTA software^34^ (version 1.26.0), specifying the —mlma command for mixed linear model association analyses on the imputed data.

Heritability is the portion of phenotypic variance due to genetic factors. Narrow-sense heritability (${\hbox {h}}^2$), which we focus on here, is the variance in a phenotype attributable to additive genetic variance i.e. that specified by a simple allele dosage model. Heritability can be estimated by partitioning the observed variation in a phenotype (e.g. varying measures of $Re_{pm}$ between individuals) into unobserved genetic and environmental factors. Traditionally, heritability is estimated by regressing offspring phenotype values onto mean parental values. This can be extended to estimate pedigree-based heritability, by analysing phenotype correlations between pairs of blood relatives, using the expected resemblance of the known pedigree structure (the kinship coefficient) in the absence of any genotype information^33^. However, this analysis tends to be low-powered if there are relatively few controls; all founders in a family must be unrelated, sharing no alleles identical by descent. Therefore, we also present a complementary heritability estimate, using a linear mixed model (LMM) approach to estimate SNP-based heritability partitioned by measured SNPs. LMM models within-family correlations to separate fixed-effect factors (e.g. gender or age) from random-effects (genomic and environmental factors unique to each individual). A key assumption of heritability estimates is that they depend on the population under study whenever genetic or environmental factors are population-specific, for instance allele frequencies or diet^35^. The statistical properties of heritability analysis methods are well described elsewhere^36^.

## Results

### Summary of participants included

Summary statistics of the 341 participants from 108 British Caucasian families are outlined in Table 3. The mean age of the cohort was 48 years old (19–73 years old) and 49% of the participants were male. 39% of the participants were hypertensive. Approximately 10% were not genotyped and therefore excluded from GWAS and SNP-based heritability estimates.

**Table 3:** Summary statistics of 341 participants from 108 British Caucasian families that underwent CMR imaging.

Trait	Mean (SD)
BMI	25.88 (3.77)
Weight (kg)	75.14 (13.50)
Clinical systolic blood pressure*	139.71 (21.83)
Clinical diastolic blood pressure*	85.07 (13.64)

The mean and range of each trait is shown. (*) not adjusted for medication intake. *SD* standard deviation.

### Reynolds number and velocity metrics

The average values of velocity recorded were ${\overline{U_{pm}}} =0.450 \, \hbox {m/s}$ and ${\overline{U_{max}}} = 0.793 \, \hbox {m/s}$ for the cross-section averaged velocity and the max value respectively, as shown in Table 4. These values are in agreement with measures obtained in previous studies^37^. The mean $Re_{pm}$ for 302 participants after outliers were removed was 4,097 (range of 1,064–7,480). 48% of participants analysed had a mean $Re_{pm}$ above 4,000, which is the threshold for turbulent flow in a straight pipe. The mean $Re_{max}$ for all samples studied was 7,328 (range of 3,288–13,588). Summary statistics of the traits can be found in Table 4.

**Table 4:** Mean and range of values for each trait and number of participants included (*n*).

Trait	n	Mean (range)
Cross sectional area (${\hbox {mm}}^2$)	317	759 (363–1,323)
Diameter (m)	319	0.031 (0.022–0.043)
$U_{pm}$ (m/s)	311	0.450 (0.110–0.800)
$U_{max}$ (m/s)	298	0.793 (0.340–1.590)
$Re_{pm}$	302	4097 (1,064–7,480)
$Re_{max}$	273	7,328 (3,288–13,588)

### Estimates of heritability

**Figure 3:**
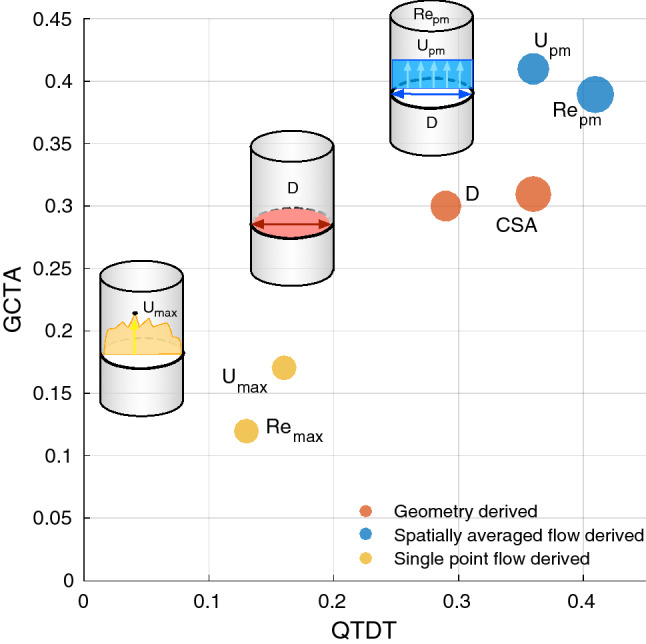
Heritability estimates for the haemodynamic metrics in the ascending aorta. Metrics are classified based on the type measurements used to calculate each: geometry based, spatially averaged flow based and single point flow based. The size of each marker is representative of the confidence level (*P* value) as presented in Table 5 where CSA is the cross sectional and D is the diameter.

Estimates of heritability for the *Re* metrics and the parameters used to calculate them, are summarised in Table 5 and presented in Fig. 3. The heritability estimates of $Re_{pm}$ suggests that 39–41% of variation in bulk maximum turbulence is due to genetic factors (*P* value = 0.0017). $Re_{max}$ was not significantly heritable; the maximum turbulence reached during the cardiac cycle is therefore mostly affected by environmental, non-genetic factors, including measurement noise.

**Table 5:** Heritability estimates of flow metrics in the ascending aorta.

Trait	QTDT ${\hbox {h}}^{2}$			GCTA SNP-based ${\hbox {h}}^{2}$		
	*P* value	h2 (%)	n	*P* value	h2 (%)	n
Cross sectional area (${\hbox {mm}}^2$)	0.0019	36	317	0.0079	31	294
Diameter (m)	0.0094	29	319	0.0101	30	295
$U_{pm}$ (m/s)	0.007	36	311	0.0020	41	287
$U_{max}$ (m/s)	$>0.05$	16	298	$>0.05$	17	276
$Re_{pm}$	0.0012	41	302	0.0017	39	279
$Re_{max}$	$>0.05$	13	273	$>0.05$	12	253

Narrow-sense heritability estimates shown here were calculated using QTDT pedigree-based software and GCTA GREML-based software. *P* values that are not significant are identified here as $>0.05$. The traits, *P* value, heritability estimate as a percentage (${\hbox {h}}^{2}$), and the sample number (n) are shown. The number of samples differs by adjustment for covariates, and 10% of samples were not genotyped so heritability estimated by GCTA had a lower sample size.

Many of the parameters involved in the *Re* calculations were estimated as significantly heritable. As expected, ascending aortic diameter (D), which remains constant for calculations of both $Re_{pm}$ and $Re_{max}$, was estimated as significantly heritable (${\hbox {h}}^2=29{-}30\%$; $P=0.0101$), similar to previously published estimates^38^ of 32–38%. Measurement of the cross sectional area (${\hbox {h}}^2 =31{-}36\%$; $P=0.0079$) was also substantially heritable. The velocities $U_{pm}$ and $U_{max}$, determine the difference in heritability between the *Re* metrics; $U_{pm}$ was significantly heritable at 36–41% ($P=0.007$), therefore creating a heritable $Re_{pm}$ trait, while $U_{max}$ was not significantly heritable, and its incorporation into the calculation of $Re_{max}$, resulted in a lack of significant heritability estimated for $Re_{max}$.

From the heritability estimates shown in Fig. 3, it can be seen that three distinct classes of metrics occur. The first consists of haemodynamic metrics considering a discrete point in both time and space and shows the lowest levels of heritability estimated for the metrics considered in this study. The second, consists of geometric metrics which have been included in previous heritability studies with a medium-high relative level of estimated heritability. The final class consists of haemodynamic metrics considering a discrete point in time but average across the cross-sectional area of the ascending aorta. This class demonstrates the highest estimation of heritability of the metrics included within the study. As anticipated given the numbers of participants studied, no locus attained GWAS significance ($\hbox {P} < 5\times 10^{-8}$).

## Discussion

This study provides the first estimates of heritability for measures of blood flow by Reynolds number (*Re*) in the ascending aorta at systole, in 300 individuals. Additionally, we describe features of aortic blood flow in larger numbers of participants than have been studied hitherto. $Re_{pm}$ has been previously estimated in literature in the ascending aorta of 30 young participants (20–30 years old) with a mean of approximately 4,500^9^. The average value of $Re_{pm}$ here was 4,097 (range of 1,064–7,480), which is similar to the previously reported measure. The trait $Re_{max}$ has been shown in literature in the range of 5,700–8,900 for 15 healthy participants of a case/control study^21^. The average value of $Re_{max}$ for all samples studied here was 7,328 (range of 3,288–13,588) in this larger cohort of families ascertained via a hypertensive proband.

Heritability has been estimated previously for a parameter used to estimate *Re* metrics; an echocardiographic-derived measure of the ascending aorta diameter was estimated to be 32–38% heritable in a mixed family-based cohort (n = 1,955 participants)^38^. We show here that while analysed in a smaller number of participants, the diameter of the ascending aorta is similarly heritable (${\hbox {h}}^2 = 29{-}30\%$). The cross-sectional area of the aorta demonstrated heritability comparable with that of the aortic diameter (${\hbox {h}}^2 = 31{-}36\%$). This result is expected given the close relationship between the two metrics and presented here to provide comparison with previous studies in the case of aortic diameter, while cross-sectional area is used in the calculation of $Re_{pm}$. In addition, the estimation of cross-sectional area is likely to be more accurate and repeatable since it is automatically calculated by edge detection software, while aortic diameter is measured by the operator and is more sensitive to error resulting from non-perpendicular measurement.

The heritability results confirm the hypothesis that variance in $Re_{pm}$ across a section of the ascending aorta is influenced by genetic factors (39–41%). Insignificant estimates of heritability were found for $Re_{max}$, which may be because it is influenced by non-genetic factors such imaging artefacts, which are to some extent averaged-out from the $Re_{pm}$ values. Given many participants presented turbulent flow characteristics, a Reynolds number estimate considering only one point is less likely to provide an accurate indication of the overall flow than an averaged value as shown in Fig. 1. This study confirms that when using image-derived flow quantities, time-averaged quantities are likely to offer a more robust assessment of the flow.

The principal aim of this study was to estimate the heritability of readily calculable MRI metrics. For completeness, we undertook a GWAS of the metrics but as anticipated given the number of participants available, this did not identify any individually significant loci. Significant covariates were identified for the CMR metrics, which required adjustment to evaluate the estimated heritability of the traits without the influence of underlying variation in phenotypes. As expected, sex, age, and BMI were identified as significant covariates for all CMR metrics analysed. Such covariates have been shown to associate with cardiac structure and function previously^39,40^, and in particular, regularly adjusted for in the analysis of aortic measurements^41,42^.

The existence of a substantially heritable aspect to the haemodynamic measures studied here is likely attributed to the role of DNA variants influencing the expression levels and function of proteins involved in haemodynamics, such as extracellular matrix proteins in the creation of the geometry of the vasculature, elastins involved in the elasticity of blood vessels, and protein hormones that influence cardiac output, such as adrenaline and angiotensin. The future discovery of genetic variants that influence aortic blood flow, can be used to assess the causality of the blood flow metrics involvement in CVD through Mendelian randomisation techniques. If confirmed, this may allow for further stratification of patients at particular risk.

Aneurysm progression is an example of a CVD where the presence of abnormal blood flow may have a significantly negative impact. Chronic disruption to flow, i.e. abnormal blood flow patterns, may impart damage to the local vessel wall and over time increase the risk of an event^43^. Aneurysms have been estimated to be highly heritable, with estimates of up to 77% for thoracic aortic aneurysms^44^. Familial Thoracic Aortic Aneurysm Disease (TAAD)^45^ due to single-gene Mendelian conditions, forms a significant proportion of TAAD. But, a proportion of TAAD and the majority of abdominal aortic aneurysm disease, is genetically complex^46^; the aetiological role of genetically determined abnormal flow remains unknown. We note that the methodology used here could also be applied to brain MRI images to assess relationships between basic haemodynamic metrics and subarachnoid and intercerebral haemorrhage (${\hbox {h}}^{2}$ of both 40%^47,48^). For example, a recent study has shown substantial heritability estimated for blood flow rate of the basilar artery (${\hbox {h}}^{2} = 24\%$)^49^.

Our methods for estimating haemodynamic metrics from imaging data are readily available from routine MRI data, and could, for example, be applied to the UK Biobank imaging cohort^50^. Blood flow imaging techniques to visualise the heart and greater vessels are being used to validate computational models of blood flow which can be used to investigate various cardiovascular diseases^51^ and are beginning to reach the clinic^52^. For example, CMR 4D flow imaging is enhancing our understanding of blood flow patterns in diseases, including tetralogy of Fallot^53^, aortic dissection^54^, and aortic bicuspid valve disease^55–57^.

There are several limitations to this study. The population is from a British Caucasion cohort and therefore heritability estimates may not be reflective of the estimated heritability in other non-European populations. The study is underpowered for traditional GWAS analysis, however the discovery of substantial heritability will allow for targeted analyses in a large-scale replication cohort. Patient data included within this study was collected from a pre-existing dataset and therefore constrained the scope of the study including the haemodynamic metrics that could be estimated.

The haemodynamic metrics calculated in this study are able to provide broad indications of blood flow characteristics. However, they are not sufficient on their own to provide a complete description of blood flow in the ascending aorta, in particular due to the pulsatile nature of the cardiac cycle. As a result, the haemodynamic metrics in this study should be treated as potential biomarkers for cardiovascular diseases rather than drivers behind their manifestation. An additional limitation of this work is the assumption of blood viscosity and density as $3\times 10^{-3}$ Ns/m$^{2}$ and 1,050 kg/m$^{3}$, respectively. While these are widely accepted estimates, it is likely some variation between individuals occurs. Finally, while some level of noise is expected in MRI images, any noise is likely to have a greater effect on a measurement taken at a single location rather than averaged over a given area. As such, from a practicality perspective, the $Re_{pm}$ measurements can be considered more robust.

While we have argued for its relevance in the ascending aorta, Reynolds number alone is insufficient to fully represent the unsteady dynamics of pulsatile blood flow. In future studies, additional metrics such as the Womersley number (ratio of unsteady and viscous forces) and the Strouhal number (ratio of oscillatory inertial forces and convective inertial forces) may provide a more complete description of the pulsatile nature of the flow, particularly in other regions of the vasculature.

In conclusion, haemodynamic metrics including measures of *Re* are substantially heritable in this family-based cohort. The *Re* measurements obtained here are in agreement with previously published results. The preliminary relationship assessed between genetics and flow metrics highlights a potential for identification of novel intervention strategies in patients with vascular abnormalities and chaotic blood flow.
